# New Appliances and Things Medical

**Published:** 1905-08-05

**Authors:** 


### NEW APPLIANCES AND THINGS MEDICAL.

[We shall be glad to receive, at our Office, 28 & 29 Southampton Street, Strand, London, W.C., from the manufacturers, specimens of all new preparations and appliances which may be brought out from time to time.]

### NAFTALAN.

(Russian Naftalan Company, A. and M. Zimmerman, 3 Lloyd's Avenue, E.C.)

We have received a sample of this ointment made as we believe by dissolving 2·5 to 4 per cent. of anhydrous soap in purified petroleum naphtha. With the preparation a brochure has been sent to us by Dr. Goldman, of Vienna, dealing with the recent therapeutic uses of naftalan. It appears that naftalan exerts marked antiseptic and local sedative effects; and is valuable as an application for eczema and pruritus. Naftalan is also useful as a basis for metallic ointments; it promotes the absorption of mercury. Extended use for a number of years of this substance has certainly proved it to be of value, and the most recent literature upon the subject tends to show that its properties continue to be recognised and utilised by the profession.

### COMPOUND OF HYPERICUM.

(Garrad, 144 The Parade, Leamington.)

This well-known preparation has changed its name, and Garrad's Compound of Hypericum will in the future be known as Compericum. This substance appears to be particularly useful for application to the skin in cases of threatened or actual bedsores, eczema, and any other conditions in which the continuity of the skin is broken. Ample experience testifies to the fact that the claims of Compericum are justified, and we have little doubt that it will continue to be much used in skin affections. It seems to combine an emollient and stimulant action.

